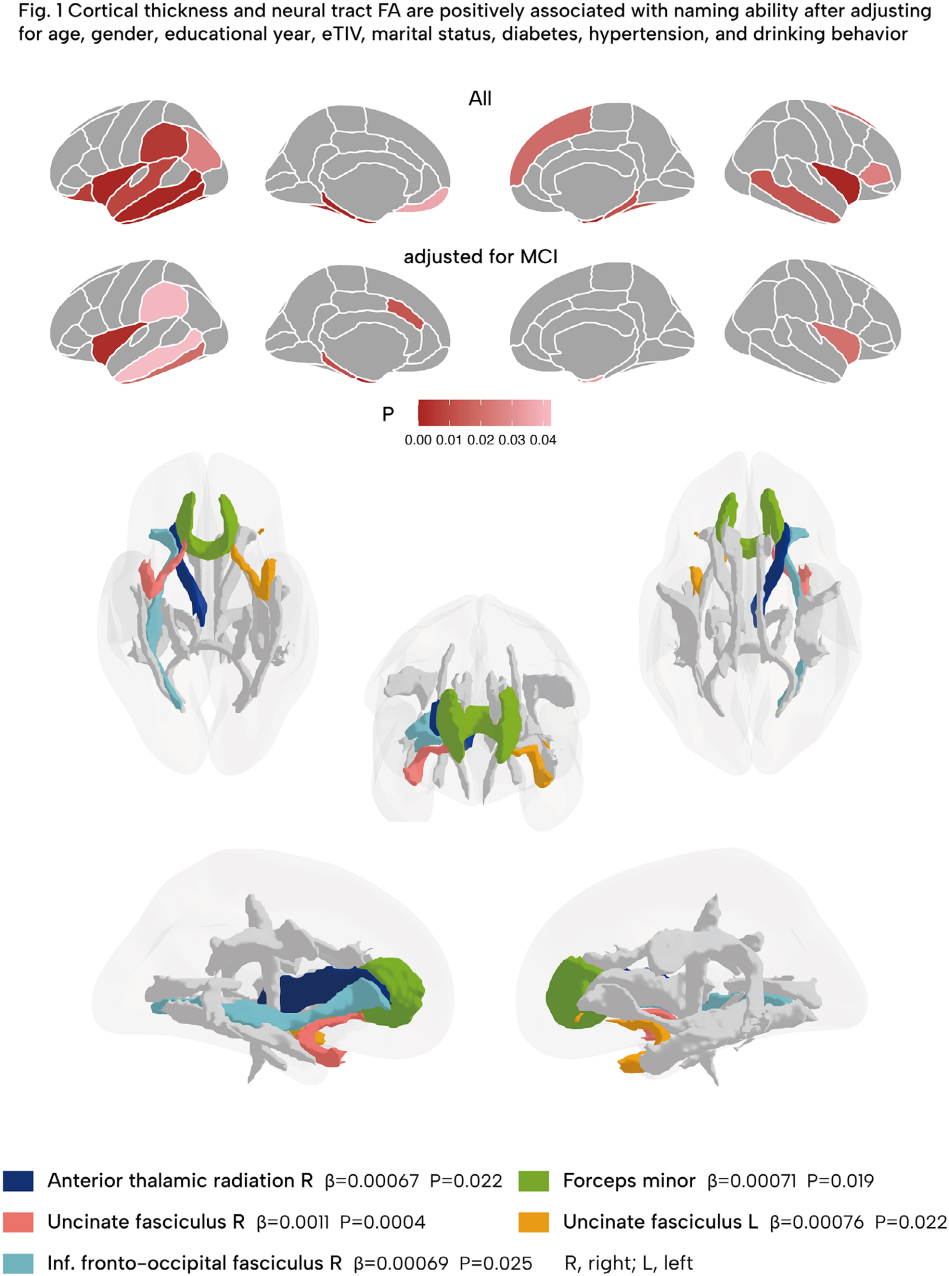# Mapping Naming Ability in the Aging Brain of Non‐Demented Older Adults: A Multimodal Neuroimaging Approach to Reveal Language Markers of Cognitive Impairment

**DOI:** 10.1002/alz70857_103211

**Published:** 2025-12-25

**Authors:** Kang‐Chen Fan, Yi‐Fang Chuang

**Affiliations:** ^1^ National Yang Ming Chiao Tung University, Taipei, Taipei, Taiwan; ^2^ National Yang Ming Chiao Tung University, Taipei, NA, Taiwan

## Abstract

**Background:**

Language impairment in Alzheimer's disease (AD) and mild cognitive impairment (MCI) has been extensively studied and considered a key early marker of AD. Recent advancements in artificial intelligence (AI) and deep learning algorithms have facilitated the development of both innovative AI‐driven and traditional language‐based tools for dementia detection and assessment. However, the neural basis underlying language production and its relationship with dementia remain inconsistent and underexplored, particularly within Mandarin‐speaking populations. This study aims to investigate the complex language system and its role in the aging process.

**Method:**

The study included 604 older adults from the Taiwan Precision Medicine Initiative on Cognitive Impairment and Dementia cohort (TPMIC), a community‐based prospective cohort. Participants underwent a comprehensive battery of neuropsychological tests, including the Boston Naming Test (BNT), and successfully completed T1‐weighted MRI, diffusion tensor imaging (DTI), and resting‐state functional MRI (rs‐fMRI). Among the participants, 112 were diagnosed with MCI, while 482 were classified as cognitively normal by an expert panel.

**Result:**

Spontaneous naming performance was significantly associated with several demographic and clinical factors, including marital status, drinking behavior, waist circumference, diabetes, hypertension, and hyperlipidemia, as well as cognitive domains such as attention, executive function, and memory. After adjusting for confounders, impaired naming ability was linked to global and regional brain atrophy, particularly in the hippocampus, entorhinal cortex, amygdala, and temporal and frontal cortices bilaterally (Figure 1).

Furthermore, white matter integrity in tracts such as the uncinate fasciculus and the inferior fronto‐occipital fasciculus was associated with naming performance. Notably, these correlations were more prominent in the right hemisphere (Figure 1), suggesting lateralized patterns that merit further investigation using rs‐fMRI data.

**Conclusion:**

Impairments in naming ability are significantly associated with other cognitive domains and brain structural changes. The observed laterality in DTI tracts may reflect unique neurodegenerative patterns in Mandarin‐speaking populations. These findings provide a foundation for the development of language‐based screening tools for dementia detection and early intervention.